# Severe Palatal Myiasis in a Young Patient With Neurological and Physical Disabilities: A Case Report

**DOI:** 10.1111/scd.70039

**Published:** 2025-05-06

**Authors:** Marcelo Santos Bahia, Yuri de Lima Medeiros, Luan Viana Faria, Alexandre Elias Trivellato, Cassio Edvard Sverzut

**Affiliations:** ^1^ Department of Oral and Maxillofacial Surgery and Periodontology Ribeirão Preto School of Dentistry University of São Paulo Ribeirão Preto São Paulo Brazil; ^2^ Department of Stomatology School of Dentistry São Paulo University São Paulo São Paulo Brazil; ^3^ Department of Diagnosis and Surgery Araraquara School of Dentistry São Paulo State University (UNESP) Ararquara São Paulo Brazil

**Keywords:** epilepsy, neurological disorders, oral myiasis, parasitic infestation

## Abstract

**Aims:**

Myiasis can be particularly debilitating in pediatric patients with neurological impairments, as communication challenges in expressing symptoms may delay diagnosis and appropriate treatment. We report a case of palatal myiasis in a young Latin American patient with neurological deficits.

**Methods and Results:**

A 16‐year‐old female with a history of severe meningitis, which resulted in neurological sequelae including spastic paralysis and epilepsy, presented with an oral lesion in the buccal cavity and episodes of fever. Upon admission, she was diagnosed with bacterial pneumonia, multiple foci of myiasis in the hard and soft palate, and dyspnea caused by larval migration to the oropharyngeal region. Larvae were manually removed, and the patient was treated with intravenous ceftriaxone, clindamycin, and ivermectin. Nitrofurazone paste was also applied topically. Four days later, surgical debridement was performed to remove necrotic mucosa from the palate. No further larvae were detected, and the patient's condition stabilized. The patient has been under follow‐up for 1 year.

**Conclusion:**

Severe palatal myiasis is a rare condition that demands prompt diagnosis and a multidisciplinary approach. This case highlights the complexity of managing myiasis in patients with physical and cognitive disabilities, especially in unfavorable socioeconomic conditions.

## 1 Introduction

Oral myiasis is a rare but significant condition in dental practice, characterized by the invasion of the oral tissues of humans by larvae of flies from the *Diptera* order. In such cases, the larvae complete their life cycle, either partially or fully, by feeding on living or necrotic tissues as well as bodily fluids [1, 2]. This condition occurs worldwide, with a higher prevalence in tropical and subtropical regions of Africa and the Americas due to the favorable warm and humid climate for the vectors [2, 3].

The incidence of oral myiasis is lower than that of cutaneous myiasis, as oral tissues are not continuously exposed to the external environment [2]. Dentists should be aware of the predisposing local factors for oral myiasis, which include poor oral hygiene, periodontal diseases, mouth breathing, anterior open bite, and tissue laceration trauma. Systemic conditions such as cognitive impairments, dementia, cerebral palsy, diabetes, and alcohol consumption further increase the risk [4, 5].

In pediatric patients or those with neurological impairments, the condition can be particularly debilitating as difficulties in communicating complaints may delay diagnosis and proper treatment [4]. In this study, we report a case of oral myiasis affecting the anterior maxillary region in a young Latin American patient with a neurological deficit. The case highlights key clinical features, diagnostic challenges, and treatment approaches, reinforcing the importance of dental professionals in identifying and managing this condition.

## 2 Case Report

### 2.1 Patient Information

A 16‐year‐old female patient, weighing 24 kg (Holliday‐Segar: 1580 mL/day), was admitted for intervention due to an oral lesion in the buccal cavity. According to her mother (caregiver), they sought medical attention approximately one week after the appearance of the lesion, which was accompanied by fever spikes of up to 39.5°C, occurring one to two episodes per day.

### 2.2 Medical History

The patient had a history of severe meningitis, which resulted in neurological sequelae, including spastic paralysis, epilepsy, and severe scoliosis. She has been dependent on a gastrostomy tube (GTT) since that time, although a previous period without the device led to severe malnutrition. The GTT was reintroduced a few years ago. The mother reported that epilepsy is currently controlled and denied frequent hospitalizations.

The family resides adjacent to a fruit and vegetable market, and the patient's room lacks insect‐proof screens. Due to her physical and cognitive impairments, the patient remains with her mouth open most of the time, combined with inadequate oral hygiene. The family has a low socioeconomic status and limited health education regarding oral care.

The patient was on continuous medications: Carbamazepine (10 mL in the morning, 20 mL at night), Valproate (Depakene, 6 mL in the morning and at night), Topiramate (125 mg in the morning and at night), and Domperidone (4 mL every 12 h).

The multidisciplinary team investigated the possibility of abuse or neglect and found no signs or history suggestive of maltreatment. Although the patient is cared for by dedicated family members, limited awareness regarding oral hygiene and early identification of myiasis was observed. Additionally, the patient's mother underwent an evaluation by the hospital psychologist, who also ruled out any indication of abuse or neglect.

### 2.3 Clinical Findings

The patient was admitted urgently and evaluated by the oral and maxillofacial surgery team as well as the medical team. She was diagnosed with pneumonia and initiated on intravenous clindamycin (40 mg/kg/day) and ceftriaxone (2 g/day). Her condition worsened with dyspnea, leading to ICU admission. Rapid IgM and IgG tests for COVID‐19 were negative. A chest computed tomography scan revealed predominant bacterial pneumonia in the left lung. The patient underwent imaging (chest CT), microbial cultures of oral secretions, blood cultures, and serological tests for HIV, TB, ANA, and VDRL, all of which returned negative results.

The bacterial pneumonia was caused by Streptococcus pneumoniae and Staphylococcus aureus, as identified in microbial cultures. Although the possibility of pulmonary myiasis coinfection was considered, it was ruled out following clinical evaluation, imaging studies, and microbiological analyses, which showed no evidence of larvae or eggs in sputum and bronchoalveolar lavage samples. The pulmonary condition was attributed to an opportunistic bacterial infection, exacerbated by the patient's clinical status.

Hematological tests indicated a preserved immune status with no evidence of immunosuppression. Clinical and laboratory evaluations revealed severe malnutrition, and the patient received nutritional support during hospitalization. The body mass index (BMI) was below the age‐appropriate percentile.

**FIGURE 1:**
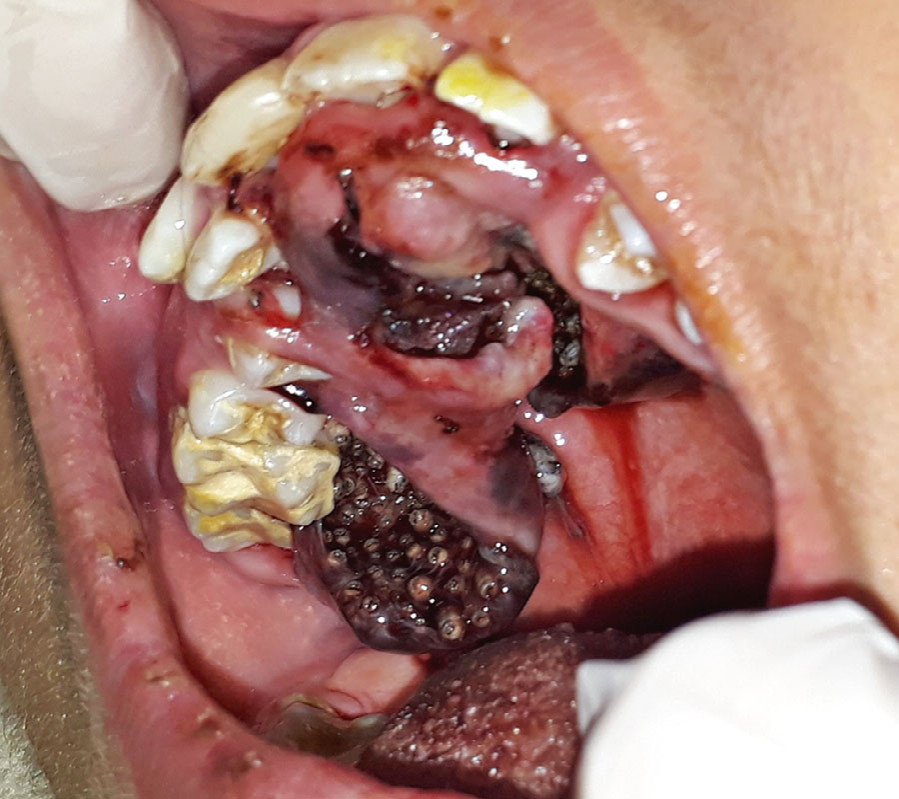
Initial appearance of the palatal lesion showing extensive infestation by myiasis larvae and eggs, highlighting the severity of the case at the time of admission.

Upon evaluation by the oral and maxillofacial surgery team, the following findings were noted: the patient was anicteric, febrile, dehydrated, non‐cyanotic, nonresponsive, eupneic, and normotensive. Multiple foci of myiasis were observed in the hard and soft palate regions (Figure 1). Additionally, respiratory difficulties were noted due to larval migration to the oropharyngeal region.

### 2.4 Therapeutic Intervention

**FIGURE 2:**
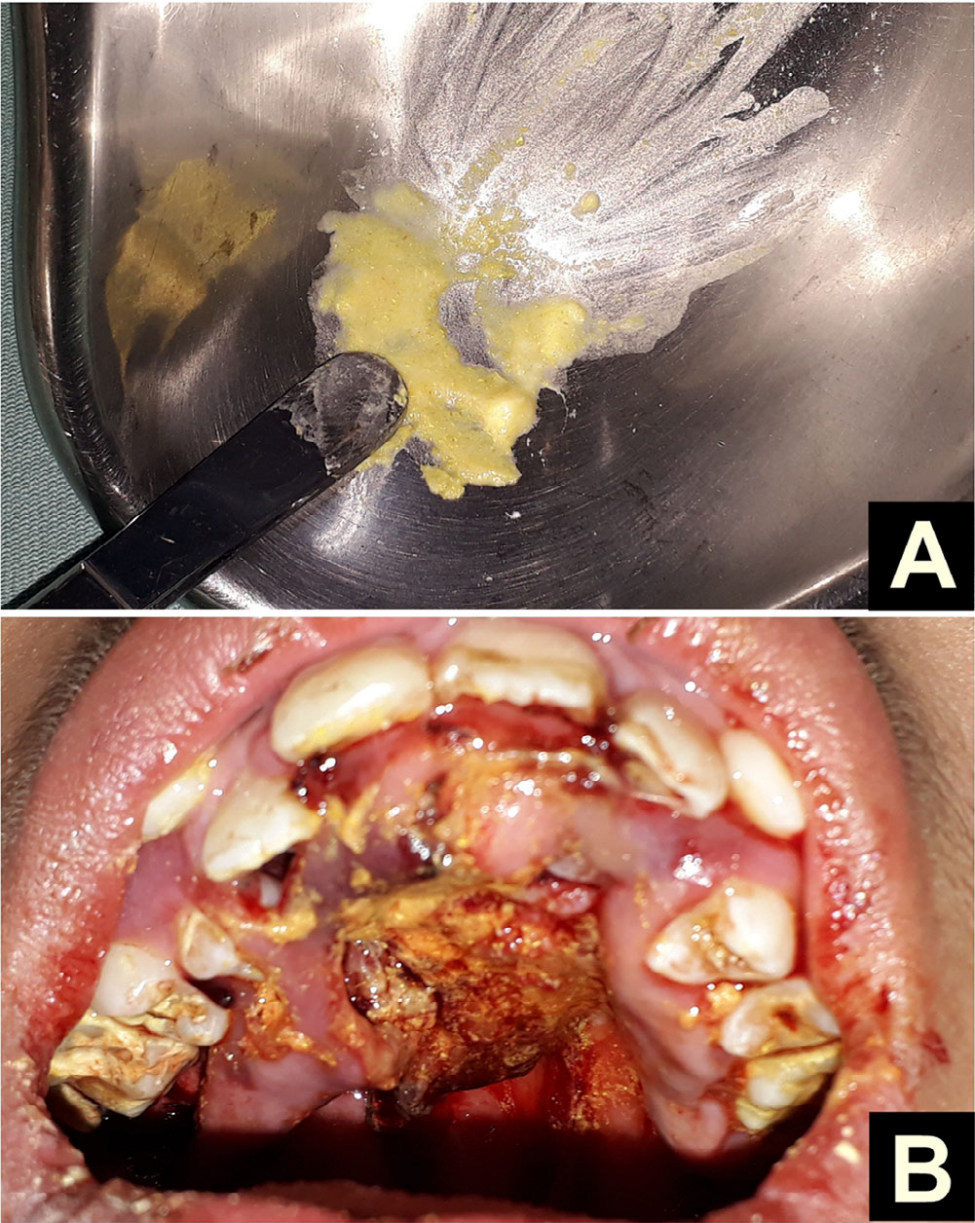
(A) Nitrofurazone paste (Furacin) prepared for local application. (B) Topical application of the paste on the palate after manual extraction of the larvae.

For the first 3 days, continuous manual extraction of larvae was performed, accompanied by irrigation with saline solution and intravenous antibiotic therapy with ceftriaxone (2 g/day) and clindamycin (40 mg/kg/day). Nitrofurazone (Furacin) paste diluted in saline was applied twice daily in the morning and afternoon (Figure 2). Intravenous ivermectin (200 µg/kg) was also administered to target any remaining eggs or larvae within the tissues.

**FIGURE 3:**
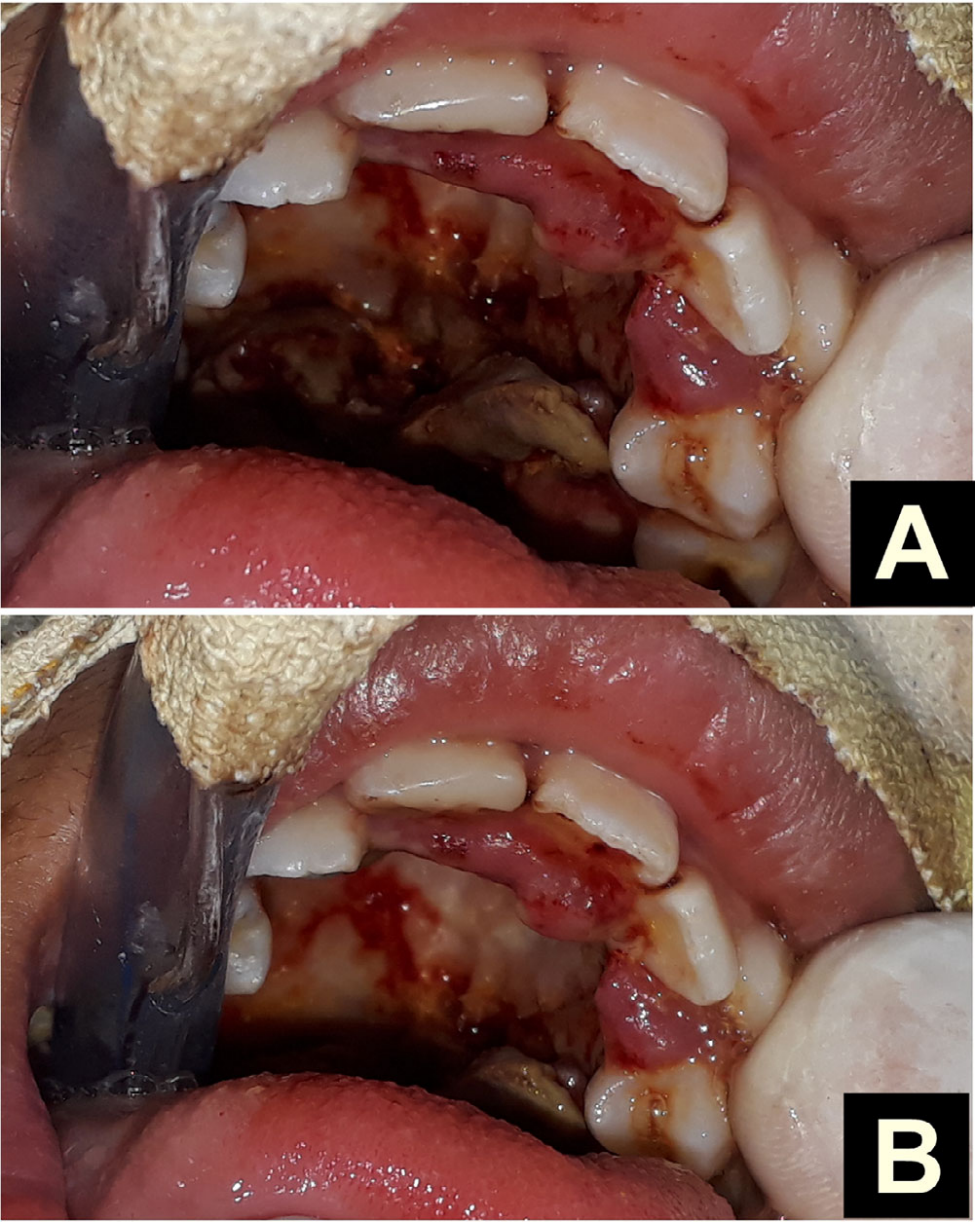
(A) Appearance of the palate on the third day after manual extraction of the larvae and treatment with Nitrofurazone (Furacin) and systemic antibiotic therapy; (B) appearance on the fifth day after surgical debridement and removal of unhealthy mucosa from the soft and hard palate.

On the fourth day, the patient underwent surgical debridement to remove unhealthy mucosa from the hard and soft palate (Figure 3). No larvae were observed during the procedure, and the patient's condition improved significantly following treatment.

After the procedures for controlling the myiasis and pneumonia, a Nutren 1.0 diet was introduced at 100% of the Holliday–Segar calculation to improve nutritional status, under the supervision of the medical and nutrition teams

### 2.5 Follow‐Up and Outcome

The patient demonstrated significant clinical improvement following multidisciplinary management, with continuous monitoring in the ICU. The combined approach of manual extraction, antibiotic therapy, and topical treatment effectively controlled the myiasis, stabilized the clinical condition, and facilitated recovery. Over the 1‐year follow‐up period, the patient exhibited no functional impairment, and complete healing of the palate occurred without bone involvement. Follow‐up visits are conducted every six months through outpatient consultations with the care team, which provides guidance on oral hygiene and basic oral healthcare. Preventive measures have been implemented to reduce the risk of recurrence, including the installation of mosquito screens at the patient's residence, adequate nutritional support from the city's nutrition team, and ongoing clinical monitoring by medical specialists.

### 2.6 Case Summary

The patient's clinical information and therapeutic aspects were summarized in Table 1.

**TABLE 1:** Clinical summary of the reported case—key clinical and therapeutic aspects.

Category	Details
**Demographic data**	16‐year‐old female
**Weight**	24 kg
**Medical history**	Severe meningitis with neurological sequelae (spastic paralysis, epilepsy, severe scoliosis), dependent on a gastrostomy tube (GTT)
**Associated conditions**	Severe malnutrition, bacterial pneumonia (Streptococcus pneumoniae and Staphylococcus aureus)
**Affected intraoral areas**	Hard and soft palate
**Diagnosis**	Oral myiasis with multiple foci and larval migration to the oropharyngeal region
**Treatment**	Continuous manual extraction of larvae for three days—Irrigation with saline solution Topical application of nitrofurazone (Furacin) Intravenous antibiotic therapy (ceftriaxone 2 g/day, clindamycin 40 mg/kg/day) Intravenous administration of ivermectin (200 µg/kg) Surgical debridement of the hard and soft palate Nutritional support (Nutren 1.0)
**Outcomes**	Complete removal of larvae Pneumonia control Full healing of the palate without bone involvement Improvement in nutritional status Continuous follow‐up with no recurrence of myiasis
**Preventive measures**	Installation of insect‐proof screens at the patient's residence Caregiver education on oral hygiene Ongoing clinical monitoring Nutritional support provided by the municipal health team

## 3 Discussion

Oral myiasis is a rare parasitic condition affecting humans and other vertebrates, caused by flies commonly found in human environments with poor hygiene and sanitation, particularly in hot and humid climates [1, 5–7]. Therefore, it has been a condition primarily documented in tropical and subtropical regions of Southeast Asia, the Americas, and Africa [4]. Infestation in humans can occur through two primary mechanisms: direct inoculation of eggs by the fly or ingestion of contaminated food, such as infected meat [2]. In the case described, the patient's cognitive condition, which results in prolonged mouth opening, combined with the lesion's location in the anterior maxilla, suggests direct inoculation of tissues by the fly.

The larvae of this family are photophobic by nature and tend to hide deep within tissues, creating a suitable environment for their development into pupae. The life cycle of a fly consists of four stages: egg, larva, pupa, and adult [2]. The requirements for egg‐laying and larval survival include moisture, necrotic tissue, and suitable temperature. Thus, wounds, open sores, crusts, and ulcers contaminated with secretions become favorable environments for larval development [1, 6]. Although no prior dental history was recorded, the oral condition observed in Figure 1, showing dental calculus accumulation, gingival inflammation, and carious lesions, indicates poor oral hygiene, making this region particularly attractive to flies. A previous systematic review has highlighted that the anterior region of the oral cavity is frequently reported as the predominant site for myiasis infestation [4].

A severely deficient nutritional status, as observed in this case, can facilitate the onset and progression of oral myiasis through multiple mechanisms. A diet lacking essential nutrients may contribute to the deterioration of oral health by disrupting tissue homeostasis, reducing resistance to microbial biofilms, and impairing tissue healing [8]. Malnourished patients often experience difficulties in mastication, hyposalivation, and compromised immune system integrity, which diminishes the body's ability to fight infections and hinders wound healing [8, 9]. These factors create an environment conducive to fly attraction and larval infestation, as open oral lesions may serve as entry points for fly larvae, further increasing the risk of oral myiasis.

Myiasis is often associated with individuals with mental disabilities [10, 11], who generally have difficulty maintaining proper oral hygiene, as observed in the case related. Shinohara et al. [11] analyzed 157 cases of oral myiasis reported in the literature over a 30‐year period and found that 18.9% of the cases were associated with neurological disorders. These patients with motor or cognitive impairments often depend entirely on their caregivers. However, caregivers usually neglect oral care in low socioeconomic settings due to a lack of health education or resources [2, 12]. A recent systematic review on oral myiasis in pediatric patients analyzed 68 reported cases and highlighted key risk factors, affected intraoral sites, treatment approaches, and preventive measures [4]. The review emphasized that children from low socioeconomic backgrounds and those with compromised hygiene and immune status are particularly vulnerable. The gingiva of the maxillary anterior region and the palate were the most commonly affected sites. This study emphasized that prevention strategies, such as hygiene education and fly control, play a crucial role in reducing the incidence of oral myiasis.

The preferred treatment for oral myiasis involves the removal of larvae combined with surgical debridement, which may or may not be supplemented with systemic medication [1, 6, 7]. Although debridement has a curative role, foreign body reactions may occur if larvae are not completely removed from the surgical wound due to incomplete debridement [1]. The use of asphyxiating agents, which induce larvae to move from deeper layers to the surface, facilitates the surgical removal procedure [6, 13].

Medications such as ivermectin and nitrofurazone have been suggested as adjuvants in treatment [4, 13, 14]. Nitrofurazone, a synthetic nitrofuran, is classified as a broad‐spectrum topical anti‐infective agent widely used in the treatment of burn patients, skin grafts, and in preventing urinary tract infections associated with catheter use [6, 14]. The administration involves its application to the wound, allowing submerged larvae to come into contact with the agent, forcing them to emerge from the tissue, and facilitating their removal. This topical treatment is typically carried out over three consecutive days and, in some cases, may obviate the need for surgical intervention. This approach is particularly advantageous in patients with severe medical conditions that contraindicate immediate surgical procedures [1, 14].

Ivermectin, on the other hand, is a synthetic antiparasitic agent with a broad spectrum of activity, belonging to the macrolide class and derived from natural substances such as avermectin, which is obtained from actinomycetes. This medication is rapidly absorbed, reaching high plasma concentrations in a relatively short time. The usual dosage is 150–200 µg/kg of body weight administered as a single dose, as indicated in this case [5, 6, 11, 13].

Early diagnosis, combined with meticulous surgical exploration of the lesion, is crucial to preventing extensive tissue damage, reducing morbidity, and avoiding the need for complex surgical repairs often required in advanced stages of the disease [1, 4]. The possibility of oral myiasis should be considered in cases of oral mucosal swelling with no apparent diagnosis, particularly in patients from regions where parasites causing myiasis are endemic. Additionally, systemic signs and symptoms such as fever and difficulty swallowing, as observed in this case, should also prompt consideration of this diagnosis.

This case highlights the complexity of managing myiasis in patients with physical and cognitive disabilities, especially in unfavorable socioeconomic conditions. Factors such as poor hygiene, an unhealthy environment, and pre‐existing neurological conditions contributed to the development of the condition. Early intervention and multidisciplinary work were crucial to avoid complications and ensure a favorable outcome.

## 4 Conclusion

Severe palatal myiasis is a rare condition that requires rapid diagnosis and an integrated approach, especially in patients with neurological problems. This report emphasizes the importance of addressing environmental and socioeconomic factors in preventing similar cases in vulnerable populations.

## Author Contributions

Marcelo Santos Bahia contributed to the study design, acquisition of data, writing of the article, proofreading of the article, and submission of the article. Yuri de Lima Medeiros contributed to the study design, writing of the article, proofreading of the article, and submission of the article. Luan Viana Faria contributed writing of the article, proofreading of the article, and submission of the article. Alexandre Elias Trivellato contributed to the planning of the study with regard to statistics, statistical analysis, proofreading of the article, study design, writing of the article, and proofreading of the article. Cassio Edvard Sverzut contributed to the planning of the study with regards to statistics, statistical analysis, proofreading of the article, study design, writing of the article, and proofreading of the article.

## Ethics Statement

All procedures followed were in accordance with the ethical standards of the responsible committee on human experimentation (institutional and national) and with the Helsinki Declaration of 1975, as revised in 2008.

## Conflicts of Interest

The authors declare no conflicts of interest.

## Consent

The informed written consent was obtained from the patient's parent to publish her photographs, radiographs, dental, and medical records.

## Data Availability

The data that support the findings of this study are available from the corresponding author upon reasonable request.
